# Women, Patriarchy and Health Inequalities: The Urgent Need to Reorient Our Systems

**DOI:** 10.3390/ijerph18094472

**Published:** 2021-04-23

**Authors:** Anna Matheson, Jacquie Kidd, Heather Came

**Affiliations:** 1 School of Health, Te Herenga Waka, Victoria University of Wellington, Aotearoa 6012, New Zealand; 2 Department of Nursing, Auckland University of Technology, Aotearoa 1010, New Zealand; jacquie.kidd@aut.ac.nz; 3 Department of Public Health, Auckland University of Technology, Aotearoa 1010, New Zealand; Heather.came@aut.ac.nz

This Special Issue is entitled “Women, patriarchy, and health inequalities: an unresolved issue”. This unresolved issue—highlighted by the COVID-19 pandemic and illustrated via this collection of articles—is the urgent need to reorient our systems away from reproducing and privileging the dominance of men through inequality, as our current systems do, and advance towards systems that privilege health and wellbeing, human rights for all and our fragile natural environment.

Globally, the pandemic has laid underlying systems of inequality within societies and between countries bare. The rich have become richer, whilst the poor and marginalised have suffered the worst health and social impacts. This is true for women globally, who are being disproportionately affected through the health, social and economic consequences of the pandemic. Most front line and essential workers are women, and it is women who have been losing their sources of income in the greatest numbers. In Aotearoa (New Zealand), for example, even with the success in containing the virus, 11,000 people lost their jobs during the March 2020 comprehensive lockdown, of whom 10,000 were women [1]. Meanwhile, in low-income countries and communities, where the virus has not been contained, women continue to have greater challenges accessing quality healthcare. These outcomes are borne from pre-existing gender inequities globally, where women bear both the greatest burden of disease and make up the largest proportion of unpaid and undervalued workers. 

Even where progress has been made when it comes to equality, and where the life expectancy of women has surpassed that of men, historical exclusion and injustices towards women continue to have far-reaching consequences because of the way our social systems are organised. One of these consequences is men’s ever-increasing economic power and control in the form of inequality, a driving force that maintains patriarchal systems through profound relationships to the social, economic, commercial and environmental determinants of health [2]. These shape who has influence in setting social priorities, and determine what our systems value. The articles in this Special Issue provide a snapshot of how current systems of power, the invisibility of women’s priorities, ideology that emphasises individuals as consumers and systemic violence shape health outcomes for women and lived experiences for all of us.

Globally, the evidence of health and inequality for women can be interpreted as both paradoxical and uneven. Some countries, despite their wealth, are still coming to terms with the need to collect health data relevant to issues that are important to women. You and colleagues [3] examine healthcare utilisation in South Korea, and Al-Hanawi and colleagues [4] explore the influences on access to breast screening in Saudi Arabia, highlighting these different stages of progress for different countries. Collecting data on health issues of significance to women is an important initial step in understanding the complexity of factors that influence women’s health outcomes. 

At the other end of the spectrum, Baum and colleagues [5] unpack the complex data-informed picture to explain why women live longer than men, even though many of the recognised social determinants of health are worse for women than for men. The authors develop an explanation for gendered life expectancy difference, confirming that, in all countries, this ranges from less than a year to over 11 years. Using in-depth case studies of the experience in Australia and Ethiopia, they demonstrate the complex socioeconomic and cultural factors that underpin these differences.

Racism worldwide compounds experiences and outcomes for women, with low-income countries carrying the burden of health inequalities [6]. Robards and colleagues [7] surveyed young people aged 12–24 years living in New South Wales, Australia, oversampling for Aboriginal and/or Torres Strait Islander; living in rural and remote areas; homeless; refugee; and/or sexuality and/or gender diversity. The authors found that the greater the extent of marginalization, the greater the negative impacts of health. Due to this, the compounding of disadvantages using an intersectional lens was found to be a useful tool to explore the outcomes for young people belonging to multiple marginalised groups.

The further marginalisation of the marginalised is also explored by Zivot and colleagues [8]. Through a scoping review, they investigated gender as a relational process necessary for the safe and healthy resettlement process of refugees in Canada. The review found that many refugee women are influenced by gender roles and expectations, as well as being exposed to gendered health systems and practices that pose risks to health, particularly mental health and access to services. They argue the need for resilience and community building to counter negative impacts of gendered beliefs and practices on health during resettlement.

Kozubik and colleagues [9] also advocate the need for community-specific solutions to address domestic violence against Roma women in Slovakia. From interviews with Roma women, the authors conclude that domestic violence results in serious psychological and physical health consequences, and that violence elimination strategies are generally set up without a specific ethnic or gender approach being taken. This can deepen the unequal position of Roma women within the family, community and society, and the acceptance of violence against Roma women. The need to account for cultural and ideological context in addressing domestic violence is reinforced by Canto and colleagues [10], who explore the experience of violence for Spanish university students.

Systems of violence and their reproduction are examined by Li and Urada [11], and Neely and colleagues [12]. In the United States, homelessness among women and the multiple vulnerabilities that they endure (such as sexual exploitation/human trafficking, violence and mental health issues) are perpetually reproduced. Through interviews, the authors found that women face a “cycle of perpetual vulnerability” with three relational pathways, these being the trauma from chronic abuse/violence inflicted on them; a state of paralysis due to inadequate availability of supportive services, shelters and mental health resources to cover all women living on the streets; and in turn, this leaves women susceptible to being a target phenotype for predators.

These cycles bear similarities to what can be considered as more “normal” experiences of women, such as pregnancy. In a study of the experience of midwives in Aotearoa (New Zealand), it was found that pregnancy increases vulnerability to poverty [12]. Women became disempowered through reduced agency, lack of opportunity and the inadequate meeting of their basic needs. Pre-existing disadvantages exacerbated risks by increasing barriers to care and causing chronic stress. The authors theorised that New Zealand’s courting with neoliberalism over the past 20 years has led to systems that emphasised individual responsibility over collective and system actions, and that despite stated aims of equitable access to healthcare for all in significant policy documents, the current system perpetuates systemic disadvantages.

This theme of emphasising individual control over the influence of the system is reinforced by Wang and Liu [13], who examined how the welfare system in China impacts women. This study focused on two major social policy branches in China—the old age pension insurance system and care services within the household. Through a gender-sensitive analysis, the authors discuss the social phenomenon of “silent reserves” (namely, women) within the Chinese welfare regime. Although women are the main contributors of long-term care and childcare, their care contributions at home are not recognized as “social achievements” and are not monetarily compensated by the patriarchal Chinese welfare state. The authors also theorise that the individualisation of care services further weakens entitlements for women, since their unpaid care constrains their ability to maintain full-time jobs in the labour market.

In exploring who benefits from individual, consumerist ideologies, Hill and Friel [14] examine how commercial interests impact the health of women and girls through corporate policies, practices and products that are increasingly affecting population health. Using the examples of the alcohol and tobacco industries, the authors argue that how they engage with women in their marketing and corporate social responsibility activities reinforces problematic gender norms and stereotypes that harm women and girls. Increasingly operating in sophisticated, multi-level ways to protect their market freedoms and their privileged position in society, these companies are able to further undermine the health of women and girls and exacerbate global health inequalities.

While we have seen substantial progress in improving the lives of women in some countries, transformative progress has not been made globally. In 2021, patriarchal systems of power still shape the lives of most women, as well as many important aspects of societies relevant to health and well-being, such as who our economies work for, and what parts of humanity and the environment are valued.

The evidence continues to paint a stark picture of the systemic undervaluing of women, their perspectives, roles and work. This is apparent in the voices within this Special Issue, which describe how our systems continue to suppress, and enact violence upon, women. To make progress when it comes to reorienting our systems away from patriarchal systems of power that are furthering inequality, we need the perspectives of women, and others who are marginalised, to hold weight and influence. A shakeup of who determines social priorities and what our systems value paves part of the pathway to a more sustainable and equitable future.
